# Supplementary material for: Methods for automatic reference trees and multilevel phylogenetic placement
Source: Bioinformatics. 2018 Aug 31;35(7):1151–8. doi: 10.1093/bioinformatics/bty767 (PMC6449752; doi:10.1093/bioinformatics/bty767)
Supplement: Supplementary Data [file bty767_supp.pdf]

Supplement:
Methods for Automatic Reference Trees and Multilevel Phylogenetic Placement

Lucas Czech, Pierre Barbera, and Alexandros Stamatakis

1 Details about the Phylogenetic Automatic (Reference) Tree Evaluation

To test the Phylogenetic Automatic (Reference) Tree (PhAT) method, we used the "SSU Ref NR 99" sequences of the Silva database (Quast *et al.*, 2013) version 123.1 and the corresponding taxonomic framework (Yilmaz *et al.*, 2014), which are available at http://www.arb-silva.de. The database contains 598 470 aligned sequences from all three domains of life, classified into 11 860 distinct taxonomic labels, and mainly contains bacterial sequences. In detail, there are

- 22 913 sequences with 347 taxonomic labels for the *Archaea*,
- 62 436 sequences with 7441 taxonomic labels for the *Eukaryota*, and
- 513 121 sequences with 4072 taxonomic labels for the *Bacteria*.

The overall number of taxonomic labels is counted here, that is, it includes higher level labels.

As explained in the main text, we constructed four sets of consensus sequences from these data using our PhAT method: a *General* set ("all of life"), as well as separate sets for the domains *Archaea*, *Bacteria*, and *Eukaryota*. The assembly of the four data sets with our method required in total about 30 min and 10 GB of memory on a standard laptop computer. This includes counting alignment characters, calculating entropies and constructing consensus sequences. The resulting data set sizes and the fraction of sequences from each domain the PhATs contain are shown in Table S1.

Our implementation of the method furthermore contains some details that are worth mentioning for reproducibility: It is possible to constrain the maximal size of clades in order to not build a consensus sequence for an overly large clade, which might not be a good representative of that clade. For the same reason, it is possible to first expand the highest ranks of the taxonomy into separate candidates. We used conservative values for these two constraints (a maximal clade size of 2000 and an expansion of only the first two taxonomic ranks), in order to give more weight to the sequence entropy. Lastly, some clades contain only one sub-clade. Those were immediately expanded, as they do not change the length of the candidate list during the algorithm.

We then inferred unconstrained and constrained maximum likelihood trees on the sequences, running 50 independent tree searches for each tree and selecting the best-scoring tree. Unconstrained trees were inferred using RAxML 8.2.8 (Stamatakis, 2014). Constrained trees were inferred with Sativa 0.9-55 (Kozlov *et al.*, 2016), which internally again relies on RAxML, and offers a convenient way to transform a taxonomy into a constraint tree.

The relative Robinson-Foulds distances (Robinson and Foulds, 1981) between the four pairs of trees (constrained versus unconstrained) are between 45.8% and 49.7%. The differences between the trees however mostly concern inner branches. As query sequences (QSs) generally tend to be placed more towards the terminal branches of the tree, the differences in the inner branches thus are acceptable for our evaluation purposes. Furthermore, we performed significance tests comparing the constrained trees to the unconstrained ones, as shown in Table S2. The tests show that in all cases, the unconstrained trees fit the data significantly better.

Given these eight PhATs, the evaluation was conducted as explained in the main text. As the sequences in Silva are already aligned to each other, no alignment step was necessary. As they contain no phylogenetic signal, we removed sites consisting entirely of gaps from the alignment, in order to reduce the memory footprint of downstream steps. Phylogenetic placement was conducted using EPA-ng (Barbera *et al.*, 2018), which is substantially faster and more scalable than RAxML-EPA (Berger *et al.*, 2011) and pplacer (Matsen *et al.*, 2010).

We evaluated the accuracy of the placements using the taxonomic labels of the sequences in Silva as an indicator of the expected branch of each sequence. Thus, we have to assume the taxonomic label of each sequence to be correct. However, errors are expected due to incongruity between the taxonomy and the phylogeny (Moreira and Philippe, 2000), as well as due to taxonomically mislabeled sequences (Kozlov *et al.*, 2016). For example, Sativa (Kozlov *et al.*, 2016), found 9934 mislabeled sequences in the Silva database. Furthermore, 17 452 sequences contain one of "incertae", "unclassified" or "unknown" in their name, indicating that those sequences might not be reliable. In total, there are 25 910 (or 4.3%) such dubious sequences in version 123.1 of the Silva database. Not all sequences are hence expected to be placed on their expected branches. We also evaluated how these dubious sequences affect tree accuracy. To this end, we used the same four trees as before (i.e., they were inferred on *all* sequences, including the dubious ones), but for the evaluation step we then excluded the dubious sequences. That is, those sequences were not placed on the trees, and their distance to the expected branch was not used for the evaluation. In most cases, this improved the results slightly (data not shown). Therefore, we decided to only report the unfiltered results.

2 Empirical Dataset Analysis

2.1 Bacterial Vaginosis

For testing the accuracy of our unconstrained *Bacteria* tree on real data, we used a vaginal microbiome dataset of 220 women (Srinivasan *et al.*, 2012), which was provided by Sujatha Srinivasan. See Figure S5 for the respective results. This small dataset with a total of 426 612 query sequences, thereof 15 060 unique, was already analyzed with phylogenetic placement methods in the original publication. We used it as an example of a well-designed study to asses our results using a PhAT as reference tree. In addition to the *Bacteria* tree, we re-inferred the reference tree of the original publication using their alignment, again using RAxML 8.2.8 (Stamatakis, 2014). The query sequences of the dataset were then aligned to our reference tree and alignment, as well as to the reference alignment of the original publication and our re-inferred tree. For aligning, we used a custom MPI wrapper of PaPaRa 2.0 (Berger and Stamatakis, 2011, 2012), which is available at (Berger and Czech, 2016). Finally, the query sequences were placed on these trees using EPA-ng (Barbera *et al.*, 2018), and the analyses were subsequently performed as explained in Figure S5.

2.2 Human Microbiome Project

We used the Human Microbiome Project (HMP) dataset (Huttenhower *et al.*, 2012; Methé *et al.*, 2012) for further testing our methods (see Figure 4). In particular, we used the "HM16STR" data of their initial phase "HMP1", which are available from http://www.hmpdacc.org/hmp/. The dataset consists of trimmed 16S rRNA sequences of the `V1V3`, `V3V5`, and `V6V9` regions. Each sample of the dataset is labeled with the human body site where it was obtained. See Table S4 for an overview of those labels. The data are further divided into a "by_sample" set and a "healthy" set, which we merged in order to obtain one large dataset, with a total of 9811 samples. We then removed 10 samples that were larger than 70 MB as well as 605 samples that had fewer than 1500 sequences, because we considered them as outliers, and finally 2 samples that did not have a valid body site label assigned to them. This resulted in a set of 9192 samples containing a total of 118 702 967 sequences with an average length of 413 bps. From these samples, sequences with a length less than 150 bps as well as sequences longer than 540 bps were removed. No further preprocessing (e.g., chimera detection) was applied. This resulted in a total of 116 520 289 sequences, of which 63 221 538 were unique. These were split into 1265 chunks of size 50 000 each, which were subsequently aligned to and placed on the unconstrained *Bacteria* tree with 2059 tips using the steps explained above. The chunk placements were then transformed again into per-sample placement files, before finally running the steps explained in Figure 4.

3 Pipeline and Implementation

An implementation of the methods described here is freely available in our tool GAPPA, which is published under GPLv3 at http://github.com/lczech/gappa. GAPPA is based on our C++ library GENESIS, which offers functionality concerning phylogenies and phylogenetic placement data, but also has functions to work with sequences, taxonomies and many other data types. GENESIS is also published under GPLv3 and is available at http://github.com/lczech/genesis.

GAPPA offers a command line interface for typical tasks of working with phylogenetic placements. The methods described in this paper are implemented via four sub-commands:

- `phat`: Phylogenetic Automatic (Reference) Tree method. The command expects a taxonomy file and a sequence file of a sequence database, e.g., SILVA (Quast *et al.*, 2013; Yilmaz *et al.*, 2014), as well as the target number of consensus sequences to be generated for the intended phylogeny. The result is a `fasta` file with consensus sequences representing taxonomic clades. The command can be further customized, e.g., by changing the consensus sequence method, using only a specified subclade of the taxonomy for running the algorithm, as well as several detail settings for the method. It can also output additional info files that allow to inspect details of the calculations, like the number of sequences and their entropy per clade.

- `extract`: Extract/collect placements in specific sub-clades of the tree. The command performs the main step of the multilevel placement approach. Its input is a set of `jplace` files containing placements on the backbone tree, as well as a file listing the clade name that each taxon of the backbone tree belongs to. For each clade, it then writes a new `jplace` file, containing all queries that were placed in that clade with more than a customizable threshold of their placement mass.
  Furthermore, if provided with the sequence files that were used to make the input `jplace` files, the corresponding sequence of each query are also written to `fasta` files per clade. Thus, a per-clade collection of sequences is created, where each result file contains the sequences that were placed in this clade of the backbone tree. These can then be used for the second level placement on separate clade-specific trees.

- `chunkify`: Split a set of `fasta` files into chunks of equal size, and write abundance maps. The command re-names the sequences using a configurable hash function (MD5, SHA1 or SHA256), and de-duplicates across all input sequences. Its output are chunk files of sequences, as well as an abundance map file for each input sequences file. The sequence chunk files can then be used to perform phylogenetic placement to obtain per-chunk `jplace` files.

- `unchunkify`: Take the per-chunk `jplace` files as well as the abundance map files, and generate a `jplace` for each original sequence file, including the correct abundances. This command is the second step of the `chunkify` command, and reverts its effect, so that the resulting `jplace` files are as if they were created using the original sequence files.

- `assign`: Perform taxonomic assignment using phylogenetic placements. While this is not the main focus of this work, we briefly introduce this method here. The command uses a taxonomic labeling of the tips of the reference tree to annotate all inner branches of the tree with the longest common taxonomic label for the induced subtree of the inner branch, in analogy to SATIVA (Kozlov *et al.*, 2016). Then, each query sequence in the provided `jplace` files is taxonomically assigned according to the labels of the branches where it does have placement mass. This can subsequently either be used for taxonomic assignment of the query sequences themselves, or to obtain a taxonomic profile of one or more samples.

These are the commands of GAPPA relevant for this paper, but it also offers more commands that are useful when working with phylogenetic placements. For details on the commands, and additional potentially useful commands, see the GAPPA documentation at https://github.com/lczech/gappa/wiki. At the time of writing, it is under active development, and more functions are planned for the near future. Furthermore, we provide prototype implementations, scripts, data and other tools used for the tests and figures in this paper at http://github.com/lczech/placement-methods-paper.

4 Taxonomic Assignment and Profiling

Taxonomic profiling is one potential application of phylogenetic placement. Thus, we also tested how well our PhATs are suited for this purpose. The CAMI Challenge (Sczyrba *et al.*, 2017) is a community-driven effort to assess taxonomic profiling methods using a common set of benchmark data sets. To assess the feasibility of using trees generated with PhAT to perform taxonomic profiling of microbiome data, we utilized the *mouse gut* data set of the 2nd CAMI Challenge (Bremges and McHardy, 2018). For this data set, we performed read preprocessing, phylogenetic placement against our constrained and unconstrained *Bacterial* trees, and subsequent taxonomic assignment.

More specifically, we used the unpaired HiSeq reads of the mouse gut data set from CAMI, which comprises 64 samples of simulated reads. The preprocessing involved read de-interleaving following Watson-Haigh (2012), paired-end read merging using PEAR (Zhang *et al.*, 2014), as well as quality filtering and conversion to `fasta` using VSEARCH2 (Rognes *et al.*, 2016). This yielded a total of 800 341 409 reads. As our trees are based on small ribosomal subunit sequences, we also performed read filtering to obtain reads from the 16S rDNA locus. This filtering was performed using the protocol of Logares *et al.* (2014), which relies on HMMER (Eddy, 1998, 2009), and respective profiles for the 16S rDNA locus. We performed a global identity based de-replication step on the resulting reads that yielded 616 405 query sequences. We aligned these query sequences to our *Bacteria* reference alignment using PaPaRa 2.0 (Berger and Stamatakis, 2011, 2012). We then performed phylogenetic placement of the aligned query sequences onto the unconstrained and constrained reference trees, respectively, using EPA-ng (Barbera *et al.*, 2018). We performed de-de-replication to obtain per-sample data again, resulting in 64 `jplace` files (one per original sample) with placements of the 16S rDNA sequences, for each of the two trees.

Finally, we performed taxonomic assignment and taxonomic profiling of the per-sample results using the `assign` command implemented in gappa, which works analogously to the method used in Sativa (Kozlov *et al.*, 2016). Its basic steps are described in Section 3. The CAMI challenge requires taxonomic assignments that conform with the NCBI taxonomy (Sayers *et al.*, 2009; Benson *et al.*, 2009). As our reference tree is however based on the Silva taxonomy (Yilmaz *et al.*, 2014), we developed a dedicated mapping procedure to, in a best effort approach, map our results to NCBI taxonomic names and IDs. The mapping is based on the *loose mapping* procedure by Balvočiūtė and Huson (2017). More specifically, we try to map taxonomic paths to their name, rank, and ID in the NCBI taxonomy, if we find a name-based match between the two. When this fails, the phylogenetic placement mass assigned to a taxonomic path by our approach is instead added to the last successful mapping further up in the taxonomic hierarchy. By initiating this procedure for each taxonomic path from its root downwards, we ensure that all placement masses are taken into account.

This mapping is a major disadvantage of our approach when using a Silva-based reference, as the Silva and NCBI taxonomies are far from being congruent (Balvočiūtė and Huson, 2017). Also note that our reference is limited to 16S rDNA locus. This substantially reduces the volume of data we can evaluate. In this particular test, only $\approx 0.08\%$ of the total data was identified as belonging to 16S. This means that our taxonomic profiling only uses a small fraction of the available data.

Finally, we used the CAMI evaluation tool for taxonomic profilers, OPAL (Sczyrba *et al.*, 2017), to compare our approach to competing software and the "gold standard" result for the data set. Despite the aforementioned caveats of taxonomic profiling using phylogenetic placements in the CAMI setting, we find that the performance of our approach is "middle of the field". Note that this is a comparison to tools that are dedicated to taxonomic profiling, which also typically can assign more of the available reads.

We show the most important OPAL results in Supplementary Figure S7 as well as in Supplementary Table S5. Furthermore, the full OPAL output can be accessed at http://github.com/lczech/placement-methods-paper.

Table S1: Taxonomic composition of the four PhATs. The table lists the four trees and their sizes (in number of tips), as well as how many of these tips originate from each of the three domains of life. The target size of the *General* tree was 2000 taxa, while the *Bacteria* and *Eukaryota* tree were targeting 1800 domain-specific taxa, which is approximately reached, but not exactly (underlined values). This is because the sizes of sub-clades in the taxonomy vary. Because each tip of the tree is a consensus sequence that represents the respective lowest taxonomic level, the number of available taxa is smaller than the total number of taxonomic labels in the Silva database. For example, the *Archaea* have a total of 347 taxonomic labels across all ranks, but only 248 labels at *Genus* level. Thus, the *Archaea* tree shown here represents the *Archaea* taxonomy resolved at the *Genus* level. In the three domain specific trees, we furthermore included consensus sequences at the *Phylum* level of the respective two remaining domains, in order to make sure that the evaluation also works well if such "outgroups" are included.

		Thereof number of		
Tree	Size	*Archaea*	*Bacteria*	*Eukaryota*
General	<u>1998</u>	210	508	1280
Archaea	511	248	205	58
Bacteria	1914	59	<u>1797</u>	58
Eukaryota	2059	59	205	<u>1795</u>

Table S2: Tree Topology Significance Tests. Here, we report significance tests comparing the four pairs of unconstrained (U) and constrained (C) trees used in our evaluation. The tests were performed with IQ-TREE v1.5.6 (Nguyen *et al.*, 2015) under the GTR+G model and 10 000 resamplings using the RELL method (Kishino *et al.*, 1990). The table shows that the unconstrained trees fit the data significantly better in all four cases and in all tests.
Columns are as follows. logL and deltaL: log likelihood and difference between constrained and unconstrained tree. bp: bootstrap proportion using RELL method (Kishino *et al.*, 1990). p-(W)KH: p-value of the one sided and the weighted Kishino-Hasegawa test (Kishino and Hasegawa, 1989). p-(W)SH: p-value of the (weighted) Shimodaira-Hasegawa test (Shimodaira and Hasegawa, 1999). c-ELW: Expected Likelihood Weight (Strimmer and Rambaut, 2002). p-AU: p-value of approximately unbiased (AU) test (Shimodaira, 2002).

Tree	logL	deltaL	bp	p-KH	p-WKH	p-SH	p-WSH	c-ELW	p-AU
General (U)	-725199.040		1.0	1.0	1.0	1.0	1.0	1.0	0.9987
General (C)	-731949.568	6750.528	0.0	0.0	0.0	0.0	0.0	0.0	0.0012
Archaea (U)	-131862.815		1.0	1.0	1.0	1.0	1.0	1.0	1.0000
Archaea (C)	-133110.463	1247.648	0.0	0.0	0.0	0.0	0.0	0.0	0.0000
Bacteria (U)	-405028.378		1.0	1.0	1.0	1.0	1.0	1.0	1.0000
Bacteria (C)	-412464.820	7436.442	0.0	0.0	0.0	0.0	0.0	0.0	0.0000
Eukaryota (U)	-745442.969		1.0	1.0	1.0	1.0	1.0	1.0	1.0000
Eukaryota (C)	-753944.998	8502.030	0.0	0.0	0.0	0.0	0.0	0.0	0.0000

Table S3: Overview of the PhATs and their evaluation statistics. Details of four unconstrained (U) and four constrained (C) trees are shown. "Size" is the number of leaves of the tree, that is, the number of consensus sequences that the tree was inferred from. "% Seqs." the percentage of sequences from SILVA placed on it. The *General* tree does not cover all sequences, because there are some sequences labels in the database that could not be mapped to the taxonomy. "∅ Br. Len." is the average branch length in the tree. The evaluation results are reported in the remaining columns: Average distances of the sequences to their respective expected branch are listed in numbers of branches (Discrete) and in branch length units (Continuous), as explained in the text. Furthermore, "Exp. Br. Hits" shows how often the most probable placement was placed exactly on the expected branch. Lastly, the average expected distance between placement locations (EDPL) is shown. The EDPL is the sum of the distances between the placements of a sequences weighted by their probability (Matsen *et al.*, 2010).

				Average Distance			
Reference Tree	Size	% Seqs.	∅ Br. Len.	Discrete	Continuous	Exp. Br. Hits	∅ EDPL
General (U)	1998	98.7%	0.084	0.63	0.034	85.9%	0.00058
General (C)	1998	98.7%	0.086	0.57	0.027	88.2%	0.00046
Archaea (U)	511	3.4%	0.070	0.46	0.013	86.4%	0.00038
Archaea (C)	511	3.4%	0.071	0.45	0.013	88.2%	0.00041
Bacteria (U)	1914	84.6%	0.067	1.13	0.031	77.0%	0.00095
Bacteria (C)	1914	84.6%	0.071	1.11	0.031	76.6%	0.00091
Eukaryota (U)	2059	10.0%	0.080	0.79	0.022	84.9%	0.00032
Eukaryota (C)	2059	10.0%	0.083	0.81	0.024	85.7%	0.00031

Table S4: HMP Dataset Overview. The table lists the 19 body site labels used by the Human Microbiome Project (HMP) (Huttenhower *et al.*, 2012; Methé *et al.*, 2012). We used this dataset to evaluate the applicability of typical analysis methods for phylogenetic placement using our PhATs, see Supplementary Section 2 and Figure 4 for details. In order to simplify the visualization in Figure 4, we summarized some of the labels into eight location regions, as shown in the second column. The last column lists how many samples from each body site were used in our evaluation.

Body Site	Region	Samples
Tongue Dorsum	Mouth (back)	610
Palatine Tonsils	Mouth (back)	599
Throat	Mouth (back)	638
Attached Keratinized Gingiva	Mouth (front)	600
Hard Palate	Mouth (front)	566
Buccal Mucosa	Mouth (front)	597
Saliva	Saliva	529
Supragingival Plaque	Plaque	608
Subgingival Plaque	Plaque	595
Anterior Nares	Airways	541
Left Retroauricular Crease	Skin	596
Right Retroauricular Crease	Skin	604
Left Antecubital Fossa	Skin	290
Right Antecubital Fossa	Skin	328
Stool	Stool	600
Vaginal Introitus	Vagina	292
Mid Vagina	Vagina	298
Posterior Fornix	Vagina	301
Sum		9192

Table S5: CAMI: Scores and Ranks. The table shows the scores and ranks of different tools evaluated with data from, and following the protocol of, the 2nd CAMI challenge (Sczyrba *et al.*, 2017; Bremges and McHardy, 2018). Here, we compare the taxonomic assignment and profiling conducted with our `assign` command to the tools that took part in the 2nd CAMI challenge. For this, we used the unconstrained and constrained *Bacterial* tree, which are abbreviated in the table as "PhAT (U)" and "PhAT (C)", respectively. See Supplementary Section 4 for details.
Four metrics are used in CAMI for evaluating tools: Recall (completeness), precision (purity), L1 norm error (abbreviated here as L1 NE), and Weighted Unifrac Error (abbreviated here as WUE). For each metric, the comparative scores of the tools are shown, as well as their rankings, relative to each other. Also, the total sum of scores and the total rank are shown, which add up the values of the four metrics. The procedure of the scoring and ranking is explained in detail in the Online Supplement of Sczyrba *et al.* (2017).
Despite the caveats and limitations that are explained in Supplementary Section 4, using our PhATs trees to obtain a taxonomic profile yields rankings in the middle of the field for all metrics.

	Total		Recall		Precision		L1 NE		WUE	
Tool	Sum	Rank	Score	Rank	Score	Rank	Score	Rank	Score	Rank
Metaphlan	1814	1	958	3	75	1	731	2	50	1
MetaPhyler	2995	2	314	1	2119	7	322	1	240	5
CommonKmers	3333	3	1448	6	409	2	1318	7	158	3
mOTU	3751	4	1703	8	488	3	1260	5	300	6
**PhAT (C)**	3784	5	1202	4	1116	4	1280	6	186	4
**PhAT (U)**	3933	6	1436	5	1153	5	1208	4	136	2
TIPP	4263	7	892	2	2126	8	930	3	315	7
FOCUS	6153	8	2079	9	1636	6	2018	8	420	8
Quikr	6838	9	1488	7	2398	9	2453	9	499	9

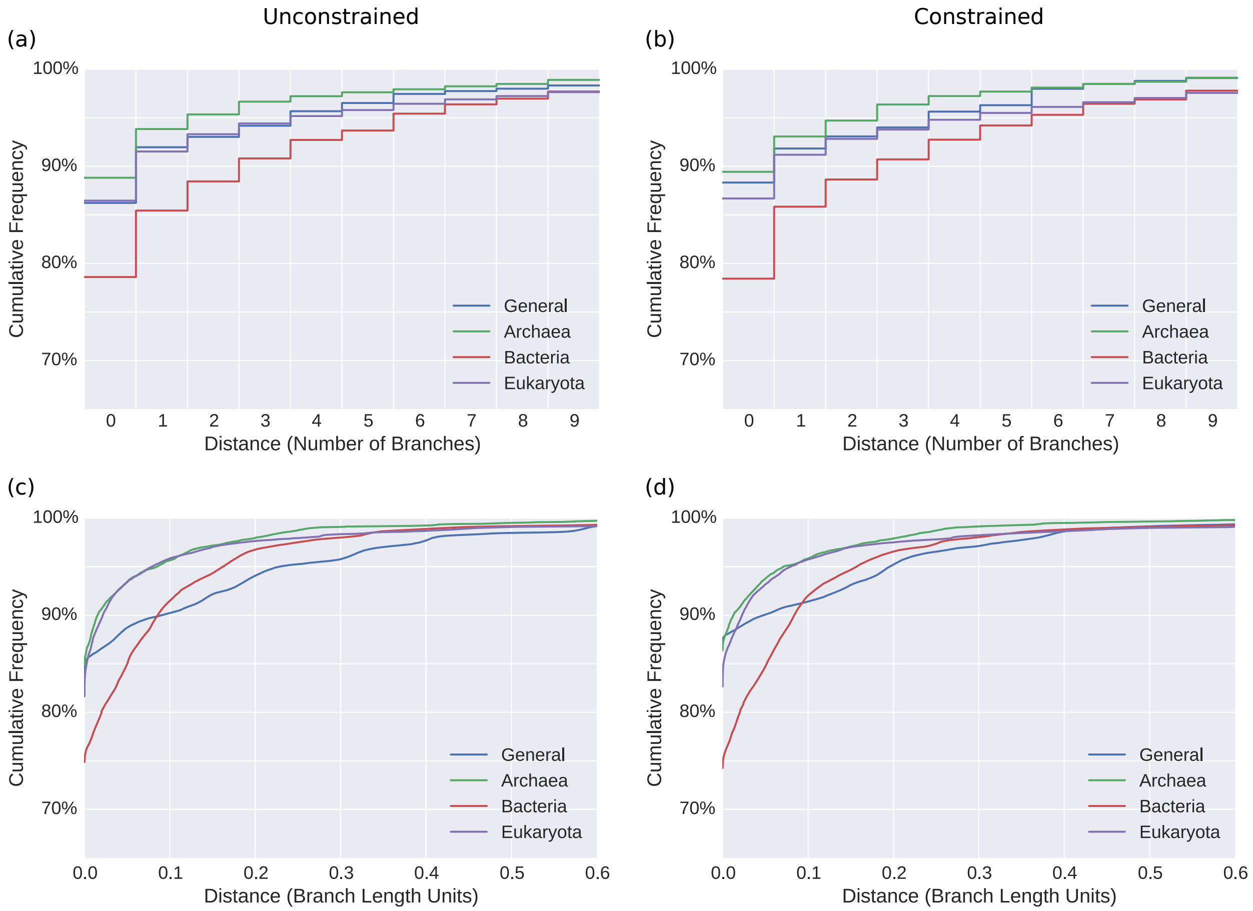

Figure S1: Accuracy comparison between unconstrained and constrained trees. Here, we compare how a taxonomic constraint changes the weighted distances to correct edges when placing our evaluation sequences on PhATs. The evaluation method is explained in the main text. Subfigures (a) and (c) are identical to Figure 3 of the main text and included here for ease of comparison. Subfigures (b) and (d) show the results when using the SILVA taxonomy as constraint for the tree inference. The relative Robinson-Foulds distances (Robinson and Foulds, 1981) between the four pairs of trees range between 45.8% and 49.7%. The differences probably occur because our trees span diverse clades, whose ancient branches are hard to resolve. Also, single gene data might not be sufficient to resolve these clades. See Supplementary Section 1 and Table S2 for a more detailed comparison of the constrained and unconstrained trees. However, the differences mostly concern inner branches. Most of the placements are however expected to be near the terminal branches, which are more stable across the trees.
This is confirmed by the fact that, overall, the results are similar between the unconstrained and constrained trees. A slight improvement can be observed for the constrained General tree (blue), which performs better according to both distance measures. However, when considering only the distance of the most likely placement (highest LWR) to its correct edge instead of using average distances weighted by the LWR per QS, the constrained trees consistently yield better results (data not shown). For example, the most significant change is observed for the Eukaryota tree, with 84% correct placements for the unconstrained tree, but 89% for the constrained one. We suspect that this is an artifact of our evaluation process, as we consider a sequence to be correctly placed if the placement branch belongs to the consensus sequence to which the sequence contributed. As the selection of sequences for each consensus sequence is guided by the taxonomy, using the same taxonomy as constraint for the tree thus might also improve the placement accuracy.

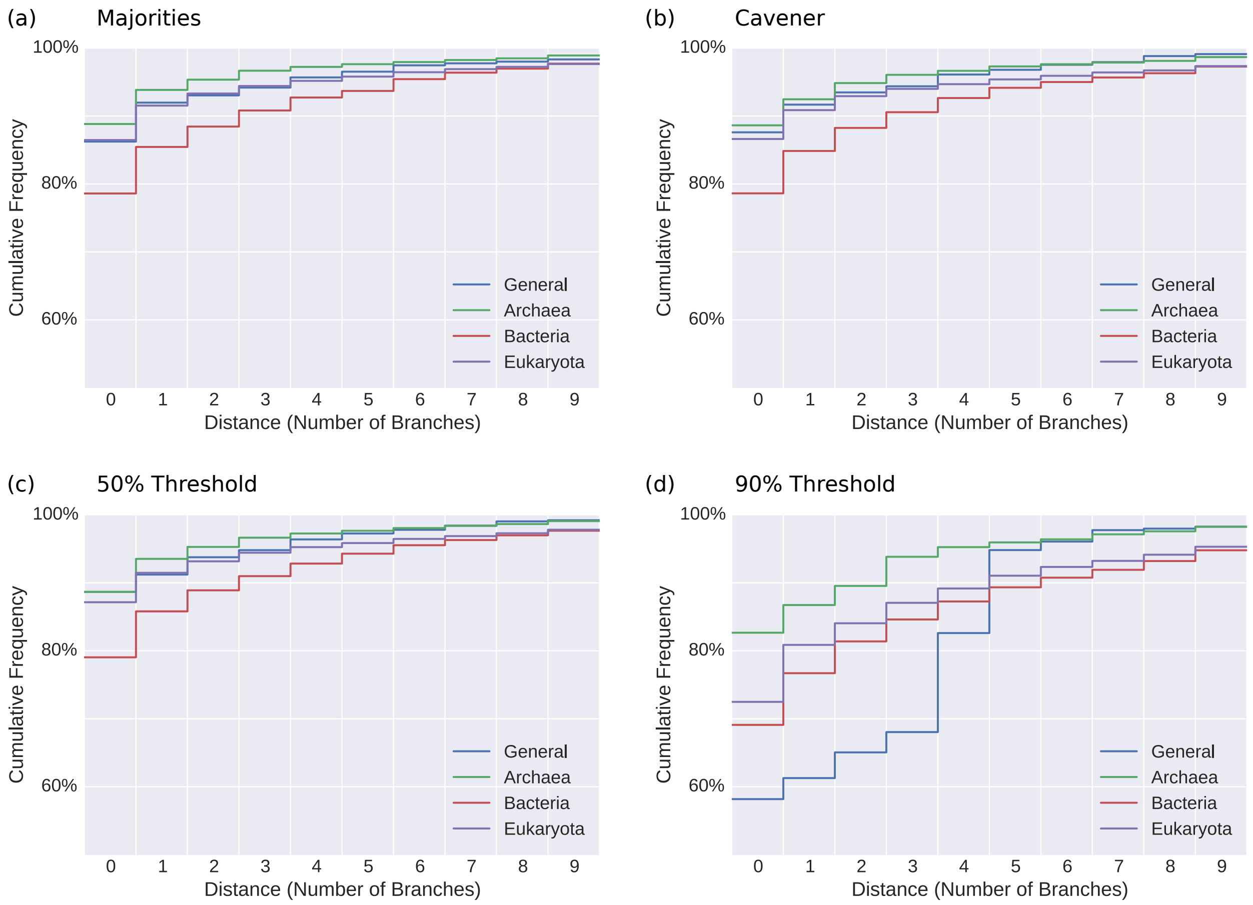

Figure S2: Effect of different consensus sequence methods on placement accuracy. In the main evaluation of our PhAT method, we used a reference tree and alignment based on majority rule consensus sequences (May, 1952; Day and McMorris, 1992a) of the Silva database sequences. Here, we evaluate the effect of using other consensus sequence methods on phylogenetic placement accuracy. The evaluation method is described in the main text. In addition to (a) majority rule consensus, we tested (b) Cavener's method (Cavener, 1987; Cavener and Ray, 1991), as well as threshold consensus sequences (Day and McMorris, 1992a,b) using thresholds of 50%, 60%, 70%, 80%, and 90%, of which two are shown in (c) and (d). The three remaining threshold methods exhibit accuracies almost exactly in between the shown plots, that is, accuracy decreases with increasing thresholds. For comparison, we also included Figure 3(a) of the main text again, here as Subfigure (a), using the same y-axis scaling as the other plots. We only show distances measured in number of branches here, because this is more relevant in the context of our methods (e.g., Multilevel Placement).

By using alternative consensus methods, the consensus sequences and thus the sites in the alignments change. Hence, the obtained reference trees (not shown) differ substantially from each other. Across the corresponding trees of the consensus methods tested here, we observed an average relative RF distance of 49.5%. This is similar to our findings depicted in in Figure S1. Here too, the accuracy of the constrained variants of these trees (data not shown) does not change much compared to the accuracy obtained for the unconstrained trees shown here. Thus, the differences in accuracy seen here are most likely due to the interplay of alignment and placed sequences (which is what we are interested in), and not due to differences in the trees (which are not of interest here).

The first three plots (a)-(c) exhibit similar accuracies. On average, majority rule, Cavener's, and low threshold ($\leq$ 70%) consensus methods place 82-83% of the sequences on the expected branch. As a general trend, the *Archaea*, being the smallest tree, tend to have the highest accuracy. On the other hand, the *Bacteria*, having the most sequences in Silva, score worst. This changes for high consensus thresholds. At high thresholds, many sites contain ambiguity characters, thus blurring the phylogenetic signal. The *General* tree, representing the highest diversity, is most affected by this.

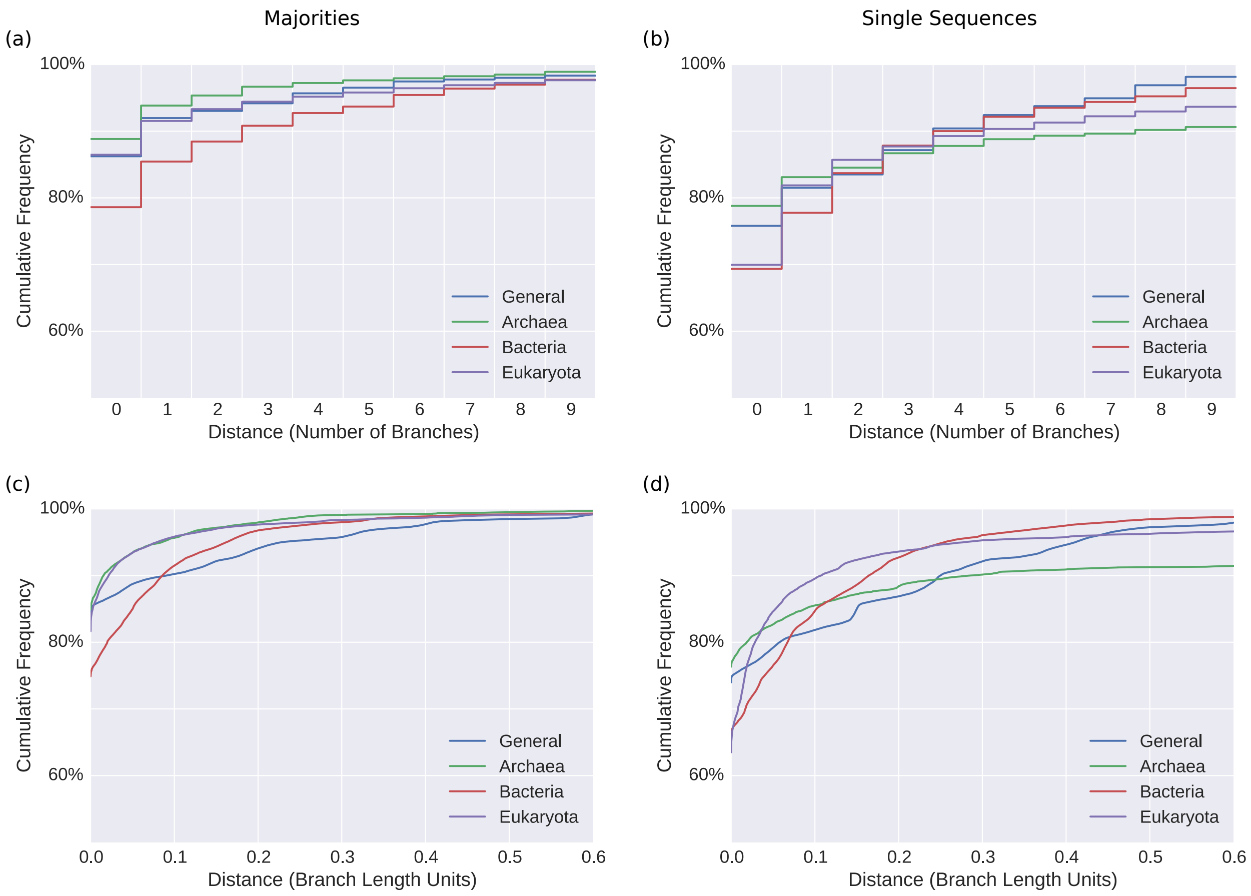

Figure S3: Effect of using actual sequences (instead of consensus sequences) on placement accuracy. For most of our evaluation of the PhAT method, we used some form of consensus sequence representation for the clades of the taxonomy, see, for instance, Figures S1 and S2. However, we also tested how the method behaves when using actual sequences instead, thus avoiding a potentially fuzzy phylogenetic signal, and related potential drawbacks of consensus sequences.

As manually selecting representative sequences from the database was not practical, we used the following automated approach. First, we took the 90% threshold consensus sequences of the PhAT method that were already evaluated in Figure S2(d). By using a high threshold, most of the diversity of a clade is represented. Then, for each such consensus sequence, we calculated a score for all sequences from the database that were used to construct this consensus sequence. This score is the number of different nucleotides between the consensus sequence and the database sequence. The sequence with the lowest score (that is, with most matching nucleotides) was then used as clade representative. Thereby, the taxonomic clades are represented by actual sequences from the database. However, as these sequences are close to the respective consensus sequence, they are still good representatives of the diversity of the clade. Using this set of sequences, we again inferred a tree and conducted the evaluation procedure by placing all sequences of the database on that tree, as described in the main text.

Subfigures (a) and (c) are identical to Figure 3 of the main text and included here for ease of comparison, however with the y-axis scaled to fit the remaining subfigures. That is, they show the evaluation of the majority rule consensus sequences. Subfigures (b) and (d) show the evaluation of the approach as described here. The resulting accuracy is worse in all cases. That is, on average, the sequences were placed further from their respective expected branch. We suspect that this is because single sequences do not capture the diversity of their clade as well as consensus sequences, and because they do not incorporate as much biological information (e.g., in form of ambiguity characters).

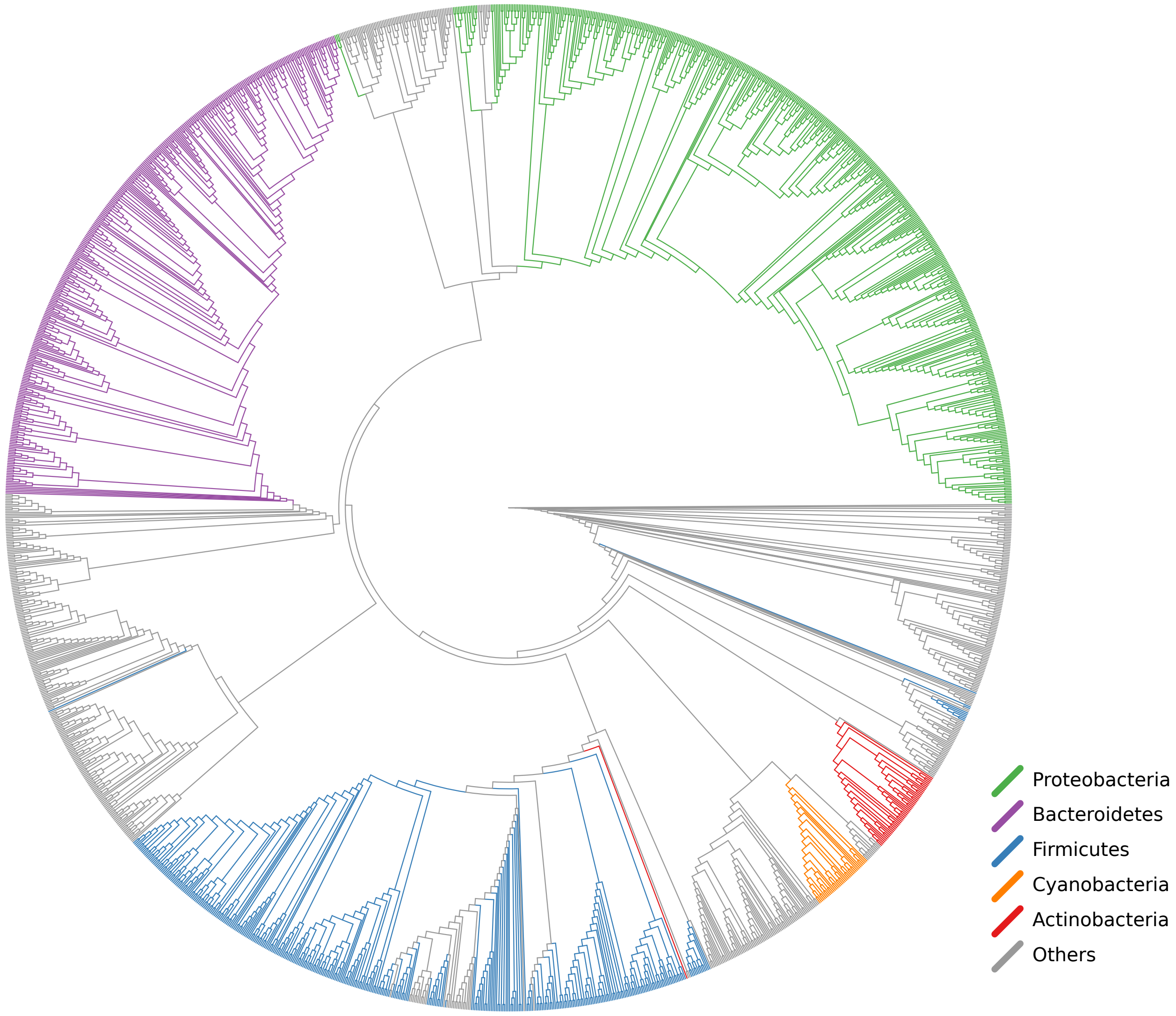

Figure S4: Unconstrained *Bacteria* tree with five bacterial sub-clades. This tree is the result of our PhAT method applied to the *Bacteria* sequences in Silva. The tree contains a total of 1914 taxa. Colorized are the five *Phylum* level sub-clades that we used for testing multilevel placement: *Proteobacteria* (505 taxa), *Bacteroidetes* (362 taxa), *Firmicutes* (360 taxa), *Cyanobacteria* (39 taxa) and *Actinobacteria* (53 taxa). The incongruence between taxonomy and phylogeny is visible here as non-monophyletic colored branches. We thus here define a clade to consist of all branches that are part of a monophyletic split of the tree with respect to the taxa in the clade. In other words, all branches on one side of a split are considered to belong to a clade, if that side of the split only contains taxa from that clade. These branches then receive the same color here. Then, for multilevel placement, a sequence is considered to be part of a clade if its most probable placement falls into that clade. For example, a sequence that is placed onto one of the orange branches on this tree is subsequently placed in the *Cyanobacteria* tree for the second level placement. Each of the five sub-clades is represented by multiple branches here, which we call the "overlap" with the *Bacteria* tree.

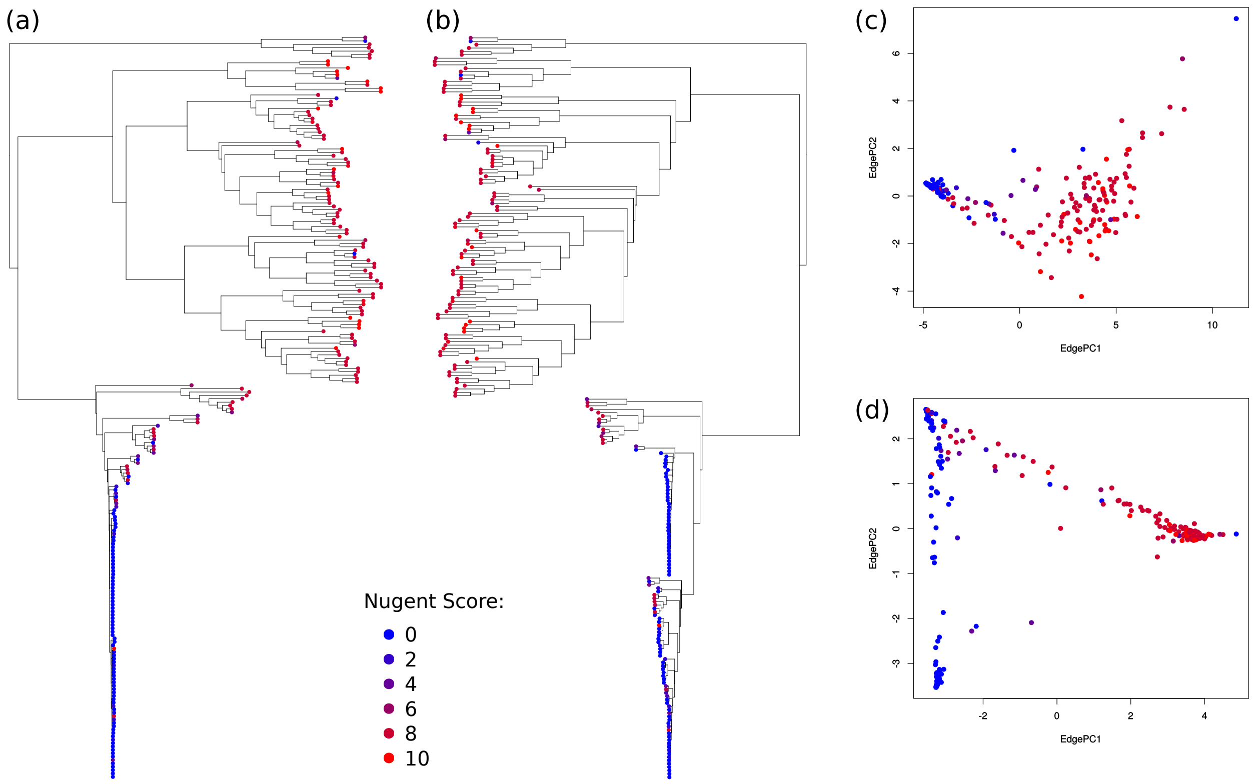

Figure S5: Assessment of a PhAT for conducting Squash Clustering and Edge PCA. Here, we test the behavior of the unconstrained *Bacteria* tree obtained via our PhAT method when used for standard post-analyses of phylogenetic placement results. For this, we used a sequence dataset of the vaginal microbiome of 220 women (Srinivasan *et al.*, 2012). For details on the processing, see Supplementary Section 2. The original study showed associations between the presence of certain bacterial species and the diagnosis of Bacterial Vaginosis (BV), a condition caused by changes in the vaginal microbiome. In the study, the Nugent score (Nugent *et al.*, 1991) was used as a clinical diagnostic criterion for BV, which ranges from 0 (healthy) to 10 (severe illness). We placed the sequences of the dataset on their original tree and on our PhAT, and reproduce some of the results from the original study to assess differences induced by using distinct references trees.
Squash Clustering is a hierarchical clustering method for phylogenetic placement data (Matsen and Evans, 2011) that uses the phylogenetic Kantorovich-Rubinstein (KR) distance (Evans and Matsen, 2012) to measure cluster similarity. The result of Squash Clustering is a cluster tree of samples, where samples that are similar according to the KR distance are closer to each other, with branch lengths according to that distance. The left side of the figure compares the cluster trees resulting from using (a) our tree and (b) the original reference tree. Subfigure (b) is a recalculation of Figure 1(A) of (Srinivasan *et al.*, 2012). The tips, which correspond to samples, are colored by the Nugent score of each sample, and thus indicate which women are affected by BV. The general features of the two cluster trees are comparable, indicating that our tree is able to distinguish between healthy and sick patients. However, there is a major difference in the lower half of the trees: While (b) shows some small branch lengths and even a separated sub-clade of samples with low Nugent score, these branches have a length of virtually zero in (a). As shown in (Srinivasan *et al.*, 2012), the healthy patients are divided into two classes, based on the presence of two species of *Lactobacillus*. The original reference tree contains sequences of those species, and can thus distinguish between them. Our broad *Bacteria* tree however does not have this degree of species-level resolution and thus treats them the same, yielding a negligible KR distance between the samples. Although this finding is expected, it serves as an example for the limits of our method.
This can also be seen in the right side of the figure, where we compare Edge PCA using (c) our PhAT and (d) the original reference tree. Edge PCA (Matsen and Evans, 2011) is a dimensionality reduction method for phylogenetic placement data, which can visualize differences between samples. Subfigure (d) is a recalculation of Figure 3(A) of (Srinivasan *et al.*, 2012). While our tree is able to separate samples by Nugent score, it does not exhibit the further separation of the two *Lactobacillus* species that is apparent when using the original tree. Thus, the samples with low Nugent score form one blob in (c).
This limitation can be overcome by either resolving the PhAT down to species level, or by using multilevel placement with a refined tree that, for example, contains species sequences of the relevant *Lactobacillus* clades. Note that analogous issues of deficient resolution or missing species can potentially also arise when hand-selecting reference sequences. In the end, it is the responsibility of the researcher to ensure that the selection of reference sequences is suitable for the dataset to be placed.

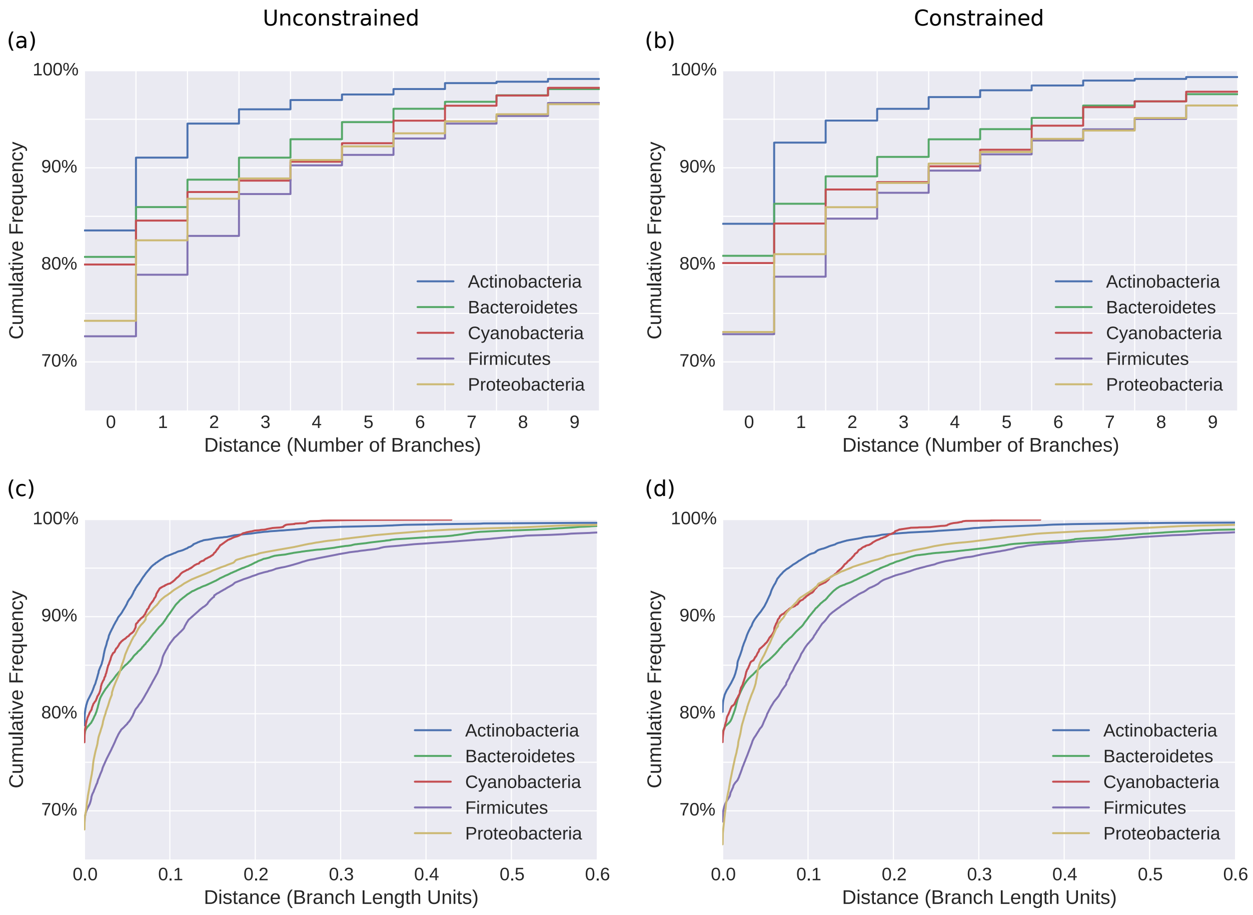

Figure S6: Accuracy of the PhAT of five bacterial sub-clades. We used five sub-clades of the *Bacteria* in SILVA, which were already scrutinized in (Kozlov *et al.*, 2016), to test how our PhAT method works for less diverse sets of sequences. These five clades are also highlighted in Figure S4; see there for a description of the clades. The evaluation was conducted as explained in the main text; in short, we placed the SILVA sequences of the clades on their respective tree, and measured how far each of them is away from the branch of the consensus sequence it is represented by.

The placement accuracy on these sub-clades is slightly worse compared to the broad *Bacteria* tree, which can be seen by comparison to Figure S1. On average, 73.4% of the sequences were placed exactly on their expected branch, dominated by *Proteobacteria* and *Firmicutes*, which combined make up 75% of the sequences in the five clades, and have an accuracy of 71%. The *Actinobacteria* have the highest accuracy, with 82% of their sequences placed on the expected branch.

The two smallest clades, *Actinobacteria* and *Cyanobacteria*, exhibit the shortest distances in branch length units. In fact, the longest distance of any sequence from its expected branch in the *Cyanobacteria* clade is around 0.4, which is indicated by the end of the red line in the lower two plots. On the other hand, the *Firmicutes* generally have the lowest accuracy here. In Figure S4, which shows the unconstrained *Bacteria* tree, the *Firmicutes* clade exhibits many paraphyletic branches, which is a known issue (Parks *et al.*, 2018). This indicates that there is a high incongruence between the *Firmicutes* taxonomy and phylogeny in SILVA, which might explain why the *Firmicutes* score worst here.

These results are likely due to the inability of 16S SSU sequences to properly resolve lower taxonomic levels (Mignard and Flandrois, 2006; Petti, 2007; Janda and Abbott, 2007). For example, Table 2 of (Janda and Abbott, 2007) lists 10 bacterial genera that are known to be hard to identify using 16S sequences. These genera account for 7.9% of the 2846 taxa that are represented by the five bacterial trees tested here. Furthermore, 95 553 of the 450 313 sequences that were placed on those trees (21.2%) belong to one of these genera. This might explain the worse scores of these clade trees. Lastly, the consensus sequences at the tips of the trees represent the *Genus* level. Thus, these have short branches, which increases the probability of misplacements.

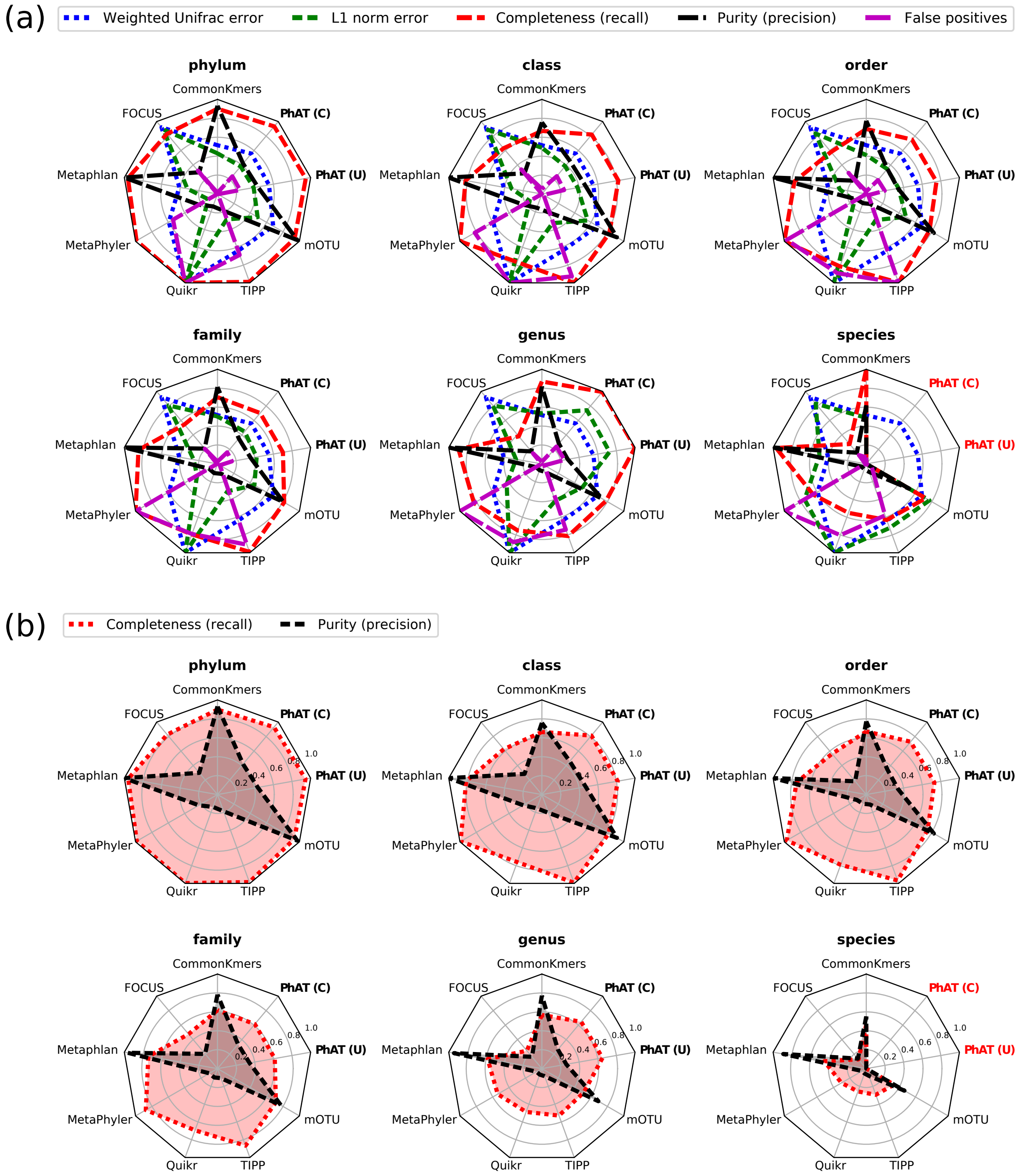

Figure S7: CAMI: Profiling Results. The figure shows the profiling results of different tools evaluated with data from, and following the protocol of, the 2nd CAMI challenge (Sczyrba *et al.*, 2017; Bremges and McHardy, 2018). Here, we compare the taxonomic assignment and profiling conducted with our `assign` command to the tools that took part in the 2nd CAMI challenge. For this, we used the unconstrained and constrained *Bacterial* tree, which are abbreviated here as "PhAT (U)" and "PhAT (C)", respectively. See Supplementary Section 4 for details on our evaluation, and see Sczyrba *et al.* (2017) for details on CAMI and their metrics.
Subfigure (a) shows the relative performance of the tools for different taxonomic ranks by displaying the error metrics used by CAMI: Weighted Unifrac error, L1 norm error, recall (completeness), precision (purity) and false positives. The error metrics were divided by their respective maximal value to allow viewing them on the same scale and to allow relative performance comparisons. Subfigure (b) shows the absolute recall (completeness) and precision (purity) for each tool across the taxonomic ranks. In both subfigures, the red text for our PhAT evaluations indicates that no predictions at the corresponding taxonomic rank were returned. This is because our Silva-based tree does not have *species* resolution and does hence not allow for taxonomic profiling at this level.

References

Balvočiūtė, M. and Huson, D. H. (2017). SILVA, RDP, Greengenes, NCBI and OTT — how do these taxonomies compare? *BMC Genomics*, **18**(2), 114.

Barbera, P. *et al.* (2018). EPA-ng: Massively Parallel Evolutionary Placement of Genetic Sequences. *bioRxiv*.

Benson, D. A. *et al.* (2009). GenBank. *Nucleic Acids Research*, **37**(Database), D26–D31.

Berger, S. and Czech, L. (2016). PaPaRa 2.0 with MPI. Online: https://github.com/lczech/papara_nt. Accessed: 2017-11-04.

Berger, S. and Stamatakis, A. (2011). Aligning short reads to reference alignments and trees. *Bioinformatics*, **27**(15), 2068–2075.

Berger, S. and Stamatakis, A. (2012). PaPaRa 2.0: A Vectorized Algorithm for Probabilistic Phylogeny-Aware Alignment Extension. Technical report, Institute for Theoretical Studies, Heidelberg.

Berger, S. *et al.* (2011). Performance, accuracy, and web server for evolutionary placement of short sequence reads under maximum likelihood. *Systematic Biology*, **60**(3), 291–302.

Bremges, A. and McHardy, A. C. (2018). Critical Assessment of Metagenome Interpretation Enters the Second Round. *mSystems*, **3**(4).

Cavener, D. R. (1987). Comparison of the consensus sequence flanking translational start sites in Drosophila and vertebrates. *Nucleic Acids Research*, **15**(4).

Cavener, D. R. and Ray, S. C. (1991). Eukaryotic start and stop translation sites. *Nucleic Acids Research*, **19**(12), 3185–3192.

Day, W. H. E. and McMorris, F. R. (1992a). Critical comparison of consensus methods for molecular sequences. *Nucleic acids research*, **20**(5), 1093–1099.

Day, W. H. E. and McMorris, F. R. (1992b). Threshold consensus methods for molecular sequences. *Journal of Theoretical Biology*, **159**(4), 481–489.

Eddy, S. R. (1998). Profile hidden Markov models. *Bioinformatics*, **14**(9), 755—-763.

Eddy, S. R. (2009). A new generation of homology search tools based on probabilistic inference. In *Genome Informatics*, volume 23, pages 205–211. World Scientific.

Evans, S. N. and Matsen, F. A. (2012). The phylogenetic Kantorovich-Rubinstein metric for environmental sequence samples. *Journal of the Royal Statistical Society. Series B: Statistical Methodology*, **74**, 569–592.

Huttenhower, C. *et al.* (2012). Structure, function and diversity of the healthy human microbiome. *Nature*, **486**(7402), 207–214.

Janda, J. M. and Abbott, S. L. (2007). 16S rRNA Gene Sequencing for Bacterial Identification in the Diagnostic Laboratory: Pluses, Perils, and Pitfalls. *Journal of Clinical Microbiology*, **45**(9), 2761–2764.

Kishino, H. and Hasegawa, M. (1989). Evaluation of the maximum likelihood estimate of the evolutionary tree topologies from DNA sequence data, and the branching order in hominoidea. *Journal of Molecular Evolution*, **29**(2), 170–179.

Kishino, H. *et al.* (1990). Maximum likelihood inference of protein phylogeny and the origin of chloroplasts. *Journal of Molecular Evolution*, **31**(2), 151–160.

Kozlov, A. M. *et al.* (2016). Phylogeny-aware identification and correction of taxonomically mislabeled sequences. *Nucleic Acids Research*, **44**(11), 5022–5033.

Logares, R. *et al.* (2014). Metagenomic 16S rDNA Illumina tags are a powerful alternative to amplicon sequencing to explore diversity and structure of microbial communities. *Environmental Microbiology*, **16**(9), 2659–2671.

Matsen, F. A. and Evans, S. N. (2011). Edge principal components and squash clustering: using the special structure of phylogenetic placement data for sample comparison. *PLOS ONE*, **8**(3), 1–17.

Matsen, F. A. *et al.* (2010). pplacer: linear time maximum-likelihood and Bayesian phylogenetic placement of sequences onto a fixed reference tree. *BMC Bioinformatics*, **11**(1), 538.

May, K. O. (1952). A set of independent necessary and sufficient conditions for simple majority decision. *Econometrica: Journal of the Econometric Society*, pages 680–684.

Methé, B. A. *et al.* (2012). A framework for human microbiome research. *Nature*, **486**(7402), 215–221.

Mignard, S. and Flandrois, J. P. (2006). 16S rRNA sequencing in routine bacterial identification: a 30-month experiment. *Journal of microbiological methods*, **67**(3), 574–581.

Moreira, D. and Philippe, H. (2000). Molecular phylogeny: pitfalls and progress. *International Microbiology*, **3**(1), 9–16.

Nguyen, L.-T. T. *et al.* (2015). IQ-TREE: A fast and effective stochastic algorithm for estimating maximum-likelihood phylogenies. *Molecular Biology and Evolution*, **32**(1), 268–74.

Nugent, R. P. *et al.* (1991). Reliability of diagnosing bacterial vaginosis is improved by a standardized method of gram stain interpretation. *Journal of clinical microbiology*, **29**(2), 297–301.

Parks, D. H. *et al.* (2018). A proposal for a standardized bacterial taxonomy based on genome phylogeny. *bioRxiv*.

Petti, C. A. (2007). Detection and identification of microorganisms by gene amplification and sequencing. *Clinical infectious diseases*, **44**(8), 1108–1114.

Quast, C. *et al.* (2013). The SILVA ribosomal RNA gene database project: improved data processing and web-based tools. *Nucleic Acids Research*, **41**(D1), D590–D596.

Robinson, D. F. and Foulds, L. R. (1981). Comparison of phylogenetic trees. *Mathematical Biosciences*, **53**(1), 131–147.

Rognes, T. *et al.* (2016). VSEARCH: a versatile open source tool for metagenomics. *PeerJ*, **4**, e2584.

Sayers, E. W. *et al.* (2009). Database resources of the National Center for Biotechnology Information. *Nucleic Acids Research*, **37**(Database), D5–D15.

Sczyrba, A. *et al.* (2017). Critical Assessment of Metagenome Interpretation—a benchmark of metagenomics software. *Nature Methods*, **14**(11), 1063–1071.

Shimodaira, H. (2002). An Approximately Unbiased Test of Phylogenetic Tree Selection. *Systematic Biology*, **51**(3), 492–508.

Shimodaira, H. and Hasegawa, M. (1999). Multiple Comparisons of Log-Likelihoods with Applications to Phylogenetic Inference. *Molecular Biology and Evolution*, **16**(8), 1114.

Srinivasan, S. *et al.* (2012). Bacterial communities in women with bacterial vaginosis: High resolution phylogenetic analyses reveal relationships of microbiota to clinical criteria. *PLOS ONE*, **7**(6), e37818.

Stamatakis, A. (2014). RAxML version 8: a tool for phylogenetic analysis and post-analysis of large phylogenies. *Bioinformatics*, **30**(9), 1312–1313.

Strimmer, K. and Rambaut, A. (2002). Inferring confidence sets of possibly misspecified gene trees. *Proceedings of the Royal Society of London B: Biological Sciences*, **269**(1487), 137–142.

Watson-Haigh, N. S. (2012). Deinterleave FASTQ files. Online: https://gist.github.com/nathanhaigh/3521724. Accessed: 2018-07-04.

Yilmaz, P. *et al.* (2014). The SILVA and "All-species Living Tree Project (LTP)" taxonomic frameworks. *Nucleic Acids Research*, **42**(D1), D643–D648.

Zhang, J. *et al.* (2014). PEAR: A fast and accurate Illumina Paired-End reAd mergeR. *Bioinformatics*, **30**(5), 614–620.
